# Integrated Assessment of Artisanal and Small-Scale Gold Mining in Ghana—Part 1: Human Health Review

**DOI:** 10.3390/ijerph120505143

**Published:** 2015-05-13

**Authors:** Niladri Basu, Edith Clarke, Allyson Green, Benedict Calys-Tagoe, Laurie Chan, Mawuli Dzodzomenyo, Julius Fobil, Rachel N. Long, Richard L. Neitzel, Samuel Obiri, Eric Odei, Lauretta Ovadje, Reginald Quansah, Mozhgon Rajaee, Mark L. Wilson

**Affiliations:** 1 Faculty of Agricultural and Environmental Sciences, McGill University, CINE Building Macdonald Campus of McGill University, 21,111 Lakeshore Rd., Ste. Anne de Bellevue, QC H9X 3V9, Canada; 2 Ghana Health Service, Accra, Ghana; E-Mails: essieclarke@yahoo.com (E.C.); odeieric@gmail.com (E.O.); 3 Department of Environmental Health Sciences, University of Michigan School of Public Health, Ann Arbor, MI 48109, USA; E-Mails: aggreen@umich.edu (A.G.); rachlong@umich.edu (R.N.L.); rneitzel@umich.edu (R.L.N.); lovadje@umich.edu (L.O.); mrajae@umich.edu (M.R.); 4 Korle Bu Teaching Hospital, Accra, Ghana; E-Mail: calys75@hotmail.com; 5 Faculty of Science, Department of Biology, University of Ottawa, ON K1N 6N5, Canada; E-Mail: laurie.chan@uottawa.ca; 6 Department of Biological, Environmental, and Occupational Health Sciences, School of Public Health, University of Ghana, Legon Boundary, Accra, Ghana; E-Mails: mdzodzo@hotmail.com (M.D.); jfobil@gmail.com (J.F.); 7 Council for Scientific and Industrial Research-Water Research Institute, Accra, Ghana; E-Mail: obirisamuel@gmail.com; 8 Noguchi Institute for Medical Research, College of Health Sciences, University of Ghana, Legon Boundary, Accra, Ghana; E-Mail: yaw121@yahoo.co.uk; 9 Department of Epidemiology, Department of Ecology and Evolutionary Biology, University of Michigan School of Public Health, Ann Arbor, MI 48109, USA; E-Mail: wilsonml@umich.edu

**Keywords:** Ghana, gold, mercury, heavy metals, occupational injuries, mining, noise, research, water, Minamata Convention

## Abstract

This report is one of three synthesis documents produced via an integrated assessment (IA) that aims to increase understanding of artisanal and small-scale gold mining (ASGM) in Ghana. Given the complexities surrounding ASGM, an IA framework was utilized to analyze economic, social, health, and environmental data, and co-develop evidence-based responses with pertinent stakeholders. The current analysis focuses on the health of ASGM miners and community members, and synthesizes extant data from the literature as well as co-authors’ recent findings regarding the causes, status, trends, and consequences of ASGM in Ghana. The results provide evidence from across multiple Ghanaian ASGM sites that document relatively high exposures to mercury and other heavy metals, occupational injuries and noise exposure. The work also reviews limited data on psychosocial health, nutrition, cardiovascular and respiratory health, sexual health, and water and sanitation. Taken together, the findings provide a thorough overview of human health issues in Ghanaian ASGM communities. Though more research is needed to further elucidate the relationships between ASGM and health outcomes, the existing research on plausible health consequences of ASGM should guide policies and actions to better address the unique challenges of ASGM in Ghana and potentially elsewhere.

## 1. Introduction

The practice of artisanal and small-scale gold mining (ASGM) is increasing in many low- and middle-income countries (LMICs) mainly due to the rising price of gold and widespread poverty. Gold from these informal mines may represent 20%–30% of the world’s output [1]. It is estimated that about 15 million people work in ASGM and that perhaps 100 million people worldwide depend on the sector for their livelihood [2]. Gold has been mined in Ghana for over 1000 years [3]; in 2012, gold accounted for 43% of the country’s national exports [4]. The proportion of Ghana’s gold that is mined through ASGM has increased from 6% in 2000 to 23% in 2010 [5].

**Figure 1:**
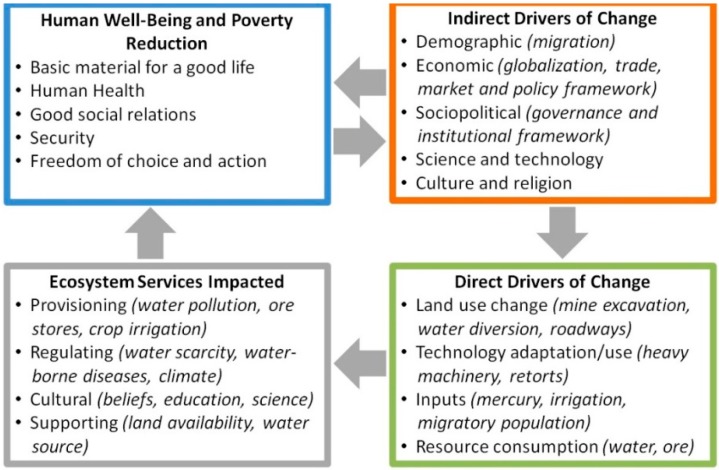
Framework linking key drivers and impacted human/natural systems. Principal domains of inquiry are highlighted. Framework is adapted from the Millennium Ecosystem Assessment [9].

Artisanal and small-scale gold mining mining, like other extractive activities, poses a number of challenges for human health [6]. Foremost is that most mining community residents are impoverished and live in rural settings that lack basic resources such as health care services and clean potable water. The United Nations Department of Economic and Social Affairs (UNDESA) describes small-scale mining as largely poverty-driven [7]. Besides the social context of these communities, mining activities ravage landscapes and contaminate ecosystems and their services [8]. The miners themselves face a number of occupational hazards, as do community members who reside in ASGM communities. Much of the scientific research to date has focused on adverse effects associated with mercury use and exposure, though there exist many additional direct and indirect factors that contribute to poor human health conditions in ASGM communities (Figure 1). This necessitates that health impacts, as well as planning for interventions and service delivery, be viewed under a broad public health lens.

### 1.1. Objective

This report is one of three studies [8,10] that are being co-published to provide a foundation for a special issue in the International Journal of Environmental Research and Public Health entitled “Integrated Assessment of Artisanal and Small-Scale Gold Mining in Ghana”. This integrated assessment (IA) is guided by an over-arching policy-relevant question: What are the causes, consequences and correctives of artisanal and small-scale gold mining in Ghana? More specifically: What alternatives are available in resource-limited settings in Ghana that allow for artisanal and small-scale gold-mining to occur in a manner that is safe for ecological health and human health without affecting near- and long-term economic prosperity? Given the complex and global nature of ASGM, an IA provides the framework for us to analyze interactions among economic, social, and environmental patterns and processes, and co-develop data-driven, evidence-based solutions with pertinent stakeholders [11]. The purpose of this report is therefore to document and scrutinize human health issues that arise from ASGM in Ghana. The ultimate goal of the endeavour is to identify response and policy options associated with ASGM in Ghana that would lead to improved human health. Ideally, the options would be sustainable, low-tech, health-promoting, and socially acceptable, while improving the health and standard of living of people who currently are involved in ASGM activities. As part of the IA, here we present evidence from Ghanaian ASGM sites that document relatively high exposures to mercury and other heavy metals, as well as health impacts through injuries and noise exposure. We also review limited data on psychosocial health, nutrition, cardiovascular and respiratory health, sexual health, and water and sanitation.

### 1.2. Limitations and Assumptions

Substantial gaps in data availability, not only in Ghana but elsewhere, prevent a full assessment of human health risks associated with ASGM. Public policy should be grounded in strong, objective, peer-reviewed science rather than anecdotes and assumptions. Speculative conclusions and opinions about possible hazards based solely upon oversimplified chronologies are not a sufficient foundation to advance regulatory reforms or policies. Nevertheless, health concerns expressed by community members and those recurring across temporal and spatial scales need to be taken seriously. In this report, all currently available evidence from Ghana, as best as possible, was reviewed and considered (peer-reviewed and non-refereed; published and non-published, *etc.*).

A majority of the evidence reviewed in this report was obtained from studies undertaken in Ghana. However, within the country there exists wide variation in the types of communities and ecosystems in which mines are situated (e.g., the South of Ghana is more tropical and economically developed than the North, and cultural differences abound across the country). While a majority of health risk factors (e.g., mercury use, poor sanitation) are ubiquitous across sites, some risks may be site-specific (e.g., content of lead or arsenic in the mined ore; local cultures and behaviours; health care services). For the purposes of this assessment we maintain broad generalizations as the focus is on developing countrywide response options.

The lack of detailed exposure assessment information for any ASGM site limits the ability to perform meaningful risk assessments and establish causal linkages. While a number of biomarker studies have been performed, as well as survey-based interviews, a rigorous and detailed exposure assessment that links source, fate, and exposure (ultimately linked to adverse health outcomes) is lacking.

The paucity of existing data also prevents us from adequately distinguishing between legal and illegal ASGM miners. In terms of operational methods, it is difficult to distinguish between the two groups of miners as they use similar methods for obtaining mineral-laden ore and for extracting the gold and other precious metals. As a result, the number of ASGM miners who operate illegally is unknown and there is great uncertainty with the estimates provided, which range from 60% to 80% [12,13].

Another limitation to understanding the human health impacts of ASGM is that they often occur on the periphery of the Ghanaian health care system, which makes surveillance data either non-existent or non-specific to miners and their families. Because of distance to health facilities, lack of familiarity with health care availability, or in some cases illegal status, ASGM participants are less likely to participate in the regular government health system. As a result, they are less often treated and missed in disease surveillance [14,15,16,17,18,19]. Furthermore, the focus of most health impacts often involves direct and observable trauma and/or short-term acute effects which are, understandably, easier to measure and document. What is often lacking are data on the longer-term adverse health outcomes that manifest as, for example, subtle loss of neurological function, reduced physical capacity, or psychological stress [15,20].

Related to these more subtle, sub-clinical, and indirect negative health effects is the complication that ‘miners’ include more than the people, mostly men, who process ore from the earth. In most ASGM settings, women and children are actively engaged in ASGM activities and are thus frequently faced with health- or life-threatening exposures [21]. In addition to individuals actively involved in mining, in ASGM areas there is often little separation between residential and mining activities. Thus community residents are often exposed to hazards that arise from ASGM. These exposures are especially difficult to identify without active surveillance and monitoring that emphasizes vulnerable groups such as women and children [19].

Finally, data may be available on diseases that might be linked to ASGM through indirect pathways that operate through social or environmental vulnerabilities, but these links are often difficult to identify and document. Identifying the ‘cause’ of some human disease events is often quite challenging if the more ‘distal’ factors are considered. For example, the indirect human health impact of ASGM on nutrition is large if crop production and food availability is diminished. Similarly, risk of malaria might be greater in ASGM communities if mosquito breeding sites are created by mining activities. Likewise, sexually transmitted diseases may become more prevalent if ASGM disrupts the social fabric of communities. The human health impacts of ASGM are therefore difficult to fully assess.

## 2. An Assessment of the Human Health Issues

**Figure 2:**
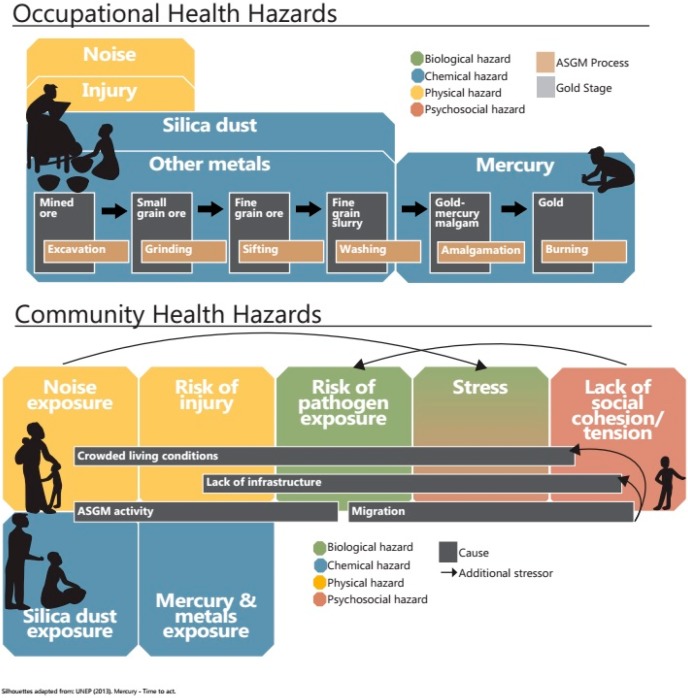
Key occupational (top panel) and community (bottom panel) human health hazards in the Ghanaian artisanal and small-scale gold mining (ASGM) sector. Silhouettes adapted from UNEP Mercury: Time to Act (2013) [22].

Here we review and analyse human health issues that arise because of ASGM in Ghana by discussing the causes, status and trends, and consequences of key hazards. This assessment summarises scientific knowledge to help build consensus and guide decision-making in the selection of response options. The information is intended to be an objective description of current conditions. We also itemize and briefly describe the key consequences of ASGM towards human health. Figure 2 displays the causes and consequences of human health hazards highlighted in this report for both occupational and community settings.

### 2.1. Mercury Exposure

#### 2.1.1. Causes

The use of mercury for gold extraction from the ore (amalgamation) is widely practiced among many small-scale miners because it is simple, inexpensive, readily available, and has a long history of use in the region. With global expansion of ASGM and ubiquitous mercury use in the sector, ASGM accounts for an estimated 37% of global atmospheric mercury emissions, and released 727 tons into the atmosphere in 2010. Additionally, ASGM is responsible for 800 tons of mercury per year released to water and land. Sub-Saharan Africa is second only to East Asia in total global emissions. Though precision and accuracy remain challenged in the aforementioned estimates, comparisons over time and space generally support that West Africa is recognised as an important source region for ASGM-related mercury emissions. Recent estimates indicate that the ASGM sector is the greatest mercury source worldwide and that mercury use in this sector continues to grow [23]. As a result of this, and other factors, ASGM has special mention in the United Nations Environment Programme (UNEP) Minamata Convention on Mercury Pollution [24].

Human exposure to mercury is complex and dictated by chemical speciation [25,26]. Mercury is found as an inorganic or organic chemical. Inorganic mercury includes elemental metallic mercury (Hg^0^, gaseous mercury) and oxidized mercury salts (Hg^+^ or Hg^2+^). In ASGM communities, exposures to elemental mercury largely occur through amalgam burning. Within the body, the kidney facilitates elimination, and mercury in urine is the accepted biomarker of recent exposure. New work using mercury stable isotopes amongst ASGM miners further supports the use of mercury in urine as a biomarker of exposures to inorganic mercury [27]. Organic mercury is generally found as methylmercury (MeHg). Exposures to MeHg largely occur through fish consumption (and in some areas, rice consumption), with the hepatobiliary system facilitating elimination and mercury in hair or blood accepted as biomarkers of exposure. “ASGM miners can be exposed to mercury via both inhalation of burned mercury and consumption of contaminated fish. However, mercury inhalation seems to be of greater relevance in Ghana given that mercury content in Ghanaian fish (even in ASGM communities) is relatively low as is fish consumption” [28]. We reviewed eight studies from Ghana that clearly demonstrate biomarker values in urine and hair above guideline values, with urinary mercury (index of elemental exposures) being similar to values found in ASGM areas throughout the world [6].

#### 2.1.2. Status and Trends

There exists strong evidence of mercury contamination in biotic and abiotic samples in proximity to ASGM sites in Ghana. As outlined in the Natural Sciences review in this series [8], mercury levels have been reported in water (range: from below detection limits to 50 µg/L), sediment (range: from below detection limits to 48.848 µg/g), soil (range: from below detection limits to 185.938 µg/g), and left over refuse or tailings (range: 0.011 to 19.296 µg/g) collected from sites across Ghana, many of which are in close proximity to mining activities. While mean mercury levels in water are below guideline values set forth by the U.S. Environmental Protection Agency (EPA) and the World Health Organization (WHO), other media show average mercury concentrations which sometimes exceed guideline values including sediment (0.17 µg/g Hg), soil (the U.S. EPA Generic Soil Screening level is 23 µg/g Hg [29]) and the Canadian Environmental Quality Guidelines are 6.6 µg/g Hg for ingestion of residential soil; 50 µg/g Hg for industrial soil [30]). In addition to these abiotic samples, mercury levels have also been found in fish and seafood (mean range: from 0.004 to 0.896 µg/g) and edible plants (mean range: 0.003 to 3.421 µg/g). Though most fish contain detectable levels of mercury, the concentrations are generally below Food and Agricultural Organization/World Health Organization (FAO/WHO) and U.S. EPA guideline values (0.5 and 0.3 µg/g, respectively) [31,32]. Generally, animal and plant species at high trophic levels show greater accumulations.

**Figure 3:**
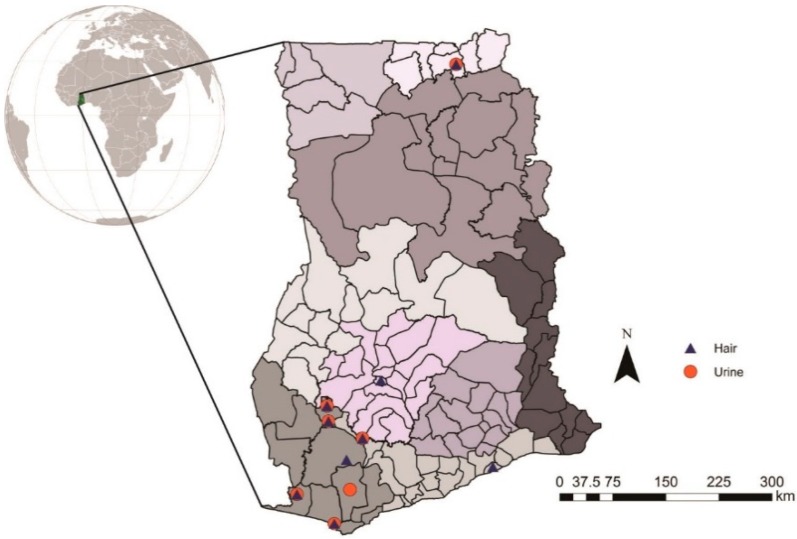
Map of sampling sites of studies on mercury in human hair and urine in Ghana. Maps produced using data provided by the United Nations Secondary Administrative Boundaries Dataset (UNSALB), by Rachel Long, July 2013.

To increase understanding of human exposures to mercury in ASGM communities, we reviewed eight studies from across Ghana. Seven of the studies were from southwest Ghana, and one was from the Upper East Region (Figure 3). Studies focusing on urine biomarkers (Table A1) and hair biomarkers (Table A2) clearly documented that all miners were exposed to appreciable mercury levels, that exposures vary greatly, and that a majority of participants have exposure biomarker levels that exceed guideline values. Within a mining site, those who exclusively burn amalgam had the highest urinary mercury levels [33]. Another important finding is that mercury levels between miners and non-miners (e.g., community members) are usually not very different, and both groups are equally exposed to relatively high levels. This is particularly of concern given that there may be upwards of ten non-mining community members for every active miner. Globally, it is estimated that 100 million people live in ASGM communities [2] and that among these individuals may be women of child-bearing age, children, and the elderly, all of whom may be especially sensitive to chemical exposures.

**Figure 4:**
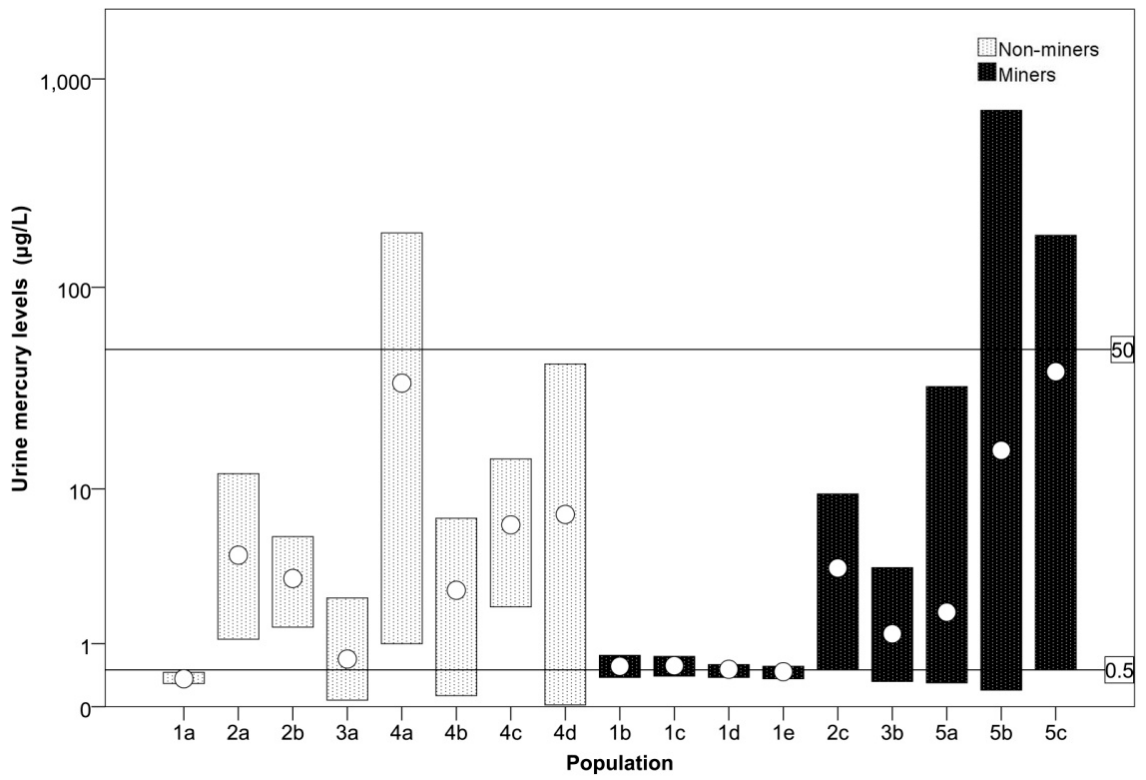
Urinary mercury levels (µg/L) from miners and non-miners across Ghana. For a given population (see key below), the bars represent the measured range while the circles represent the mean. Lines represent the WHO guideline value of 50 μg/L for occupational exposure [37], and 0.5 μg/L, the average mercury concentration in urine for the U.S. population [38]. The population numbers refer to various studies as follows. Study Population 1—Southwest Ghana in large-scale and small-scale gold mining area, [36]: 1a. Non-miners 10 km from mines (*n =* 12); 1b. permanent large-scale miners (*n =* 12); 1c. Casual large-scale miners (*n =* 12); 1d. Large-scale miners 0.5 km from mines (*n =* 12); 1e. Large-scale miners 2 km from mines (*n =* 12). Study Population 2—Tarkwa, Western Region [35]: 2a. Non-miners in mining area (*n =* 15); 2b. Non-miners in non-mining area (*n =* 4); 2c. Miners in mining area (*n =* 17). Study Population 3—Dunkwa-on-Offin, Central Region [34]: 3a. Farmers (*n =* 54); 3b. Small-scale miners (*n =* 40). Study Population 4—Ankroba and Tano River Basins, Western Region [39]:4a–4d. Mining area residents, possibly involved in mining (*n =* 50, 50, 51, 66, respectively). Study Population 5—Talensi-Nabdam District, Upper East Region [33]: 5a-c. Small-scale miners in mining areas (*n =* 13, 92, 15, respectively).

Three studies compared urine mercury levels from miners to those of non-miners. In Dunkwa-on-Offin, a study found the mean urinary mercury concentration in small-scale miners (1.23 μg/L; range: 0.32–3.62 μg/L) to be higher than that in farmers (0.69 μg/L; range: 0.08–2.31 μg/L) [34]. Asante *et al*. compared urinary mercury concentrations among Tarkwa miners (3.6 μg/L; range: 0.50–9.4 μg/L), to non-miners living in the same area (4.3 μg/L; range: 1.1–12 μg/L), and non-mining Accra residents (3.1 μg/L; range: 1.4–5.5 μg/L), and did not find much difference [35]. For comparative purposes, a study in southwest Ghana on large-scale miners documented urinary mercury in three groups of large-scale miners (means: 0.56, 0.57, 0.51 μg/L) and non-miners 10 km from the mine site (0.36 μg/L) [36]. In all of these studies, no means exceeded the WHO guideline value of 50 μg/L for occupational exposure [37], but many were above 0.5 μg/L, the average mercury concentration in urine for the U.S. population [38] (Figure 4). 

**Figure 5:**
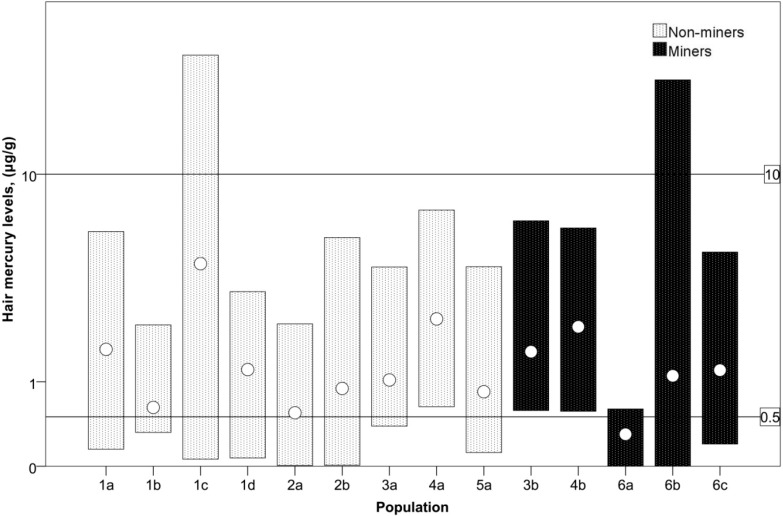
Mercury levels (µg/g) in hair samples of miners and non-miners from across Ghana. For a given population (see key below), the bars represent the measured range while the circles represent the mean. Lines represent the WHO guideline value of 10 μg/g for mercury in hair [40], and 0.5 μg/g, the average mercury concentration in hair for the U.S. population [38]. Study Population 1—Ankroba and Tano River Basins, Western Region [39]: 1a–d. Mining area residents, possibly involved in mining (*n =* 50, 50, 51, 66, respectively). Study Population 2—Accra, Greater Accra Region, and Kumasi, Ashanti Region [43]: 2a. Students from Accra; 2b. Students from Kumasi (*n =* 19, 18, respectively). Study Population 3—Pra River Basin (in Western, Central, and Ashanti Regions) [41]: 3a. Non-miners in mining area (*n =* 24); 3b. Small-scale miners (*n =* 38). Study Population 4—Dunkwa-on-Offin, Central Region [34]: 4a. Farmers (*n =* 54); 3b. Small-scale miners (*n =* 40). Study Population 5—Throughout Ghana [42]: 5a. General population (*n =* 123). Study Population 6—Talensi-Nabdam District, Upper East Region [33]: 6a–c. Small-scale miners (*n =* 11, 77, 12, respectively).

While means did not exceed the WHO guideline level, individuals likely did, as the maximum of the range in several studies was above 50 μg/L. Two studies directly compared mercury levels in hair by occupation. No mean values recorded in these studies exceeded the WHO guideline value of 10 μg/g for mercury in hair [40], but many were above 0.5 μg/g, the average mercury concentration in hair for the U.S. population [38] (Figure 5). Donkor *et al*. recorded mean hair mercury concentrations of small-scale gold miners (1.56 µg/g) and non-small-scale miners (1.03 µg/g) in southwestern communities [41]. Kwaansa-Ansah *et al*. found mean mercury concentrations in hair of small-scale gold miners (2.14 µg/g) and non-small-scale miners (2.35 µg/g) in Dunkwa-on-Offin [34]. These studies do not show differences between miners and non-miners. 

To put the aforementioned data into perspective, a study by Voegborlo *et al.* found a mean hair mercury level of 0.843 µg/g (range: 0.119–4.14 µg/g) in 123 people sampled from the general population of Ghana [42]. Anim *et al*., compared mercury concentrations in hair between students in Accra (mean: 0.551 µg/g) and students in Kumasi (0.893 µg/g) [43]. While mercury in hair is often used as a preferred biomarker of MeHg exposure in ASGM communities, its use needs to be carefully questioned given new work using mercury stable isotopes that shows that a majority of hair mercury may be derived exogenously from adsorbed inorganic mercury [27]. One study from Ghana’s Upper East region compared mercury exposure across the different kinds of jobs that comprise ASGM [33]. Workers who handle mercury had significantly higher urinary mercury levels than the other types of occupational groups, including mechanical operators, administrators of the concessions, excavators who blast and chisel ore, those who sift and grind the crushed ore, and support workers.

#### 2.1.3. Consequences

The health effects of mercury poisoning have been well documented from many populations worldwide. For example, chronic poisoning produces irritability, nervousness or excitability, insomnia, dysarthria (motor speech disorder), incoordination, and hallucinations. Acute poisoning causes dizziness, vomiting and headaches [44]. However, to our knowledge, such health effects in Ghanaian ASGM communities have yet to be documented. Beyond Ghana, studies of ASGM communities in other parts of the world have provided some evidence of mercury-associated adverse health outcomes, specifically, effects on the renal, nervous, and immune systems [6]. However, teasing apart the contributing role of mercury from the myriad of other chemical and non-chemical stressors is challenging.

#### 2.1.4. Certainty Analysis

There is high certainty (a number of scientific publications; plausibility, strength of association, consistency of findings) that both ASGM miners and community members across Ghana are exposed to high, and potentially dangerous, levels of mercury. Though technical credibility of some studies is questioned, as some used inappropriate or inadequate surveys and sampling techniques, mercury use has been associated with contamination of air, water, soil, sediments, and foodstuffs at a number of sites in Ghana. Consequently, biomarker studies have shown human contamination to be widespread in ASGM communities. Accumulation of mercury is known to cause a number of adverse health effects, though causal evidence from Ghana is limited (but expected based on the literature from elsewhere). Spatial trends are difficult to elucidate, as most data are collected in a few key mining areas in Ghana, such as Tarkwa and Obuasi, but less often in comparison areas including the general population. Only one study to our knowledge has documented mercury pollution in the Upper East [33], and no studies have been conducted in the Upper West. Temporal trends are likewise difficult to determine, as most studies on mercury in Ghana have only been conducted in the last five to ten years, and even within a site there exists considerable spatial and temporal variability in exposures.

### 2.2. Toxicant Exposures—Other Heavy Metals

#### 2.2.1. Causes

Although most human health research in ASGM communities has evaluated mercury exposure, much less is known about exposure to other potentially toxic elements. Several steps in the processing of gold-containing ore (Figure 2), such as crushing and amalgamation, may facilitate the release of other chemicals into the environment. Despite the potential for widespread contamination, there is little empirical evidence addressing this, particularly regarding human exposures. Recent poisoning events, such as the childhood mortality observed in Zamfara, Nigeria in 2010, have been attributed to lead exposure [45], yet the potential for this type of occurrence in Ghana is unknown.

#### 2.2.2. Status and Trends

In Ghana, there is some evidence of ASGM communities being exposed to multiple toxic elements, such as arsenic and cadmium (Table 1). As described in the Natural Sciences review in this series [8], researchers have documented a number of potentially toxic elements in water, food, and soil from ASGM communities. The health effects of these exposures, however, have not been directly measured and can only be indirectly inferred by analogy with findings from other studies.

In southwest Ghana, a high proportion of miners and community residents have been shown to have elevated levels of urinary arsenic [35]. In the Upper East Region, urinary metals were characterized in 57 male miners. Chromium and arsenic exceeded health guideline values for 52% and 34%, respectively, of all participants. About 10%–40% of the participants had urinary levels of aluminium, copper, manganese, nickel, selenium, and zinc that fell outside the U.S. reference ranges [46].

#### 2.2.3. Consequences

A large body of literature has established that human exposure to heavy metals is associated with several diseases. Here we focus on arsenic and cadmium as these metals have the most data available using reliable biomarkers.

Arsenic at concentrations above 50 µg/L in drinking water is associated with excess cancer risk (e.g., bladder, kidney, liver, lung, and prostrate), cardiovascular diseases, blood pressure, anemia in pregnancy, obstructive lung diseases, mortality from respiratory diseases, and diabetes in adults; and neurodevelopment problems, skin lesions, cancer, and respiratory diseases in children [47]. At concentrations around 10 µg/L in drinking water, considered safe by the World Health Organization’s provisional guideline, arsenic may also cause cancer in the order of 0.1%–0.3% and increase systolic blood pressure in women six weeks postpartum [48,49]. Inorganic arsenic easily crosses human and animal placenta and has been demonstrated to increase the risk of impaired fetal growth and fetal/infant mortality in human populations [50,51,52,53,54] and further evidenced in a systematic review and meta-analysis [55].

For cadmium, the main target of toxicity is the kidney, but it also affects the bone, cardiovascular system, and immune system. Cadmium is classified as a Class 1 carcinogen by the U.S. EPA, and has been linked to prostate, pancreatic, and lung cancer [56]. Further research is needed to determine if exposures to these elements (and others) may be affecting health in ASGM communities.

Metals known to contaminate ASGM communities may be of carcinogenic risk. Risk assessments based on heavy metal concentrations, especially arsenic, in surface and groundwater samples in Tarkwa and in cassava from four mining communities in the Western Region both found cancer risks higher than the U.S. EPA’s acceptable range [57,58]. Notably, in a case-study of two galamsey (unregistered) sites in the Upper Denkyira and Wassa West Districts, both miners and medical staff associated various cancers with mercury use in ASGM but this was not done using any rigorous statistical methods [59]. Further, IARC classifies mercury and inorganic mercury compounds as Group 3 chemicals “not classifiable as to [their] carcinogenicity to humans.” To our knowledge there exist no data on cancer rates in ASGM sites, and more work is needed in this area.

**Table 1:** Arsenic (As) and cadmium (Cd) in human urine from ASGM miners and non-miners from across Ghana.

Metal	Study Location	Population	Sample Size (n)	Mean (µg/L)	SD (µg/L)	Median (µg/L)	Min (µg/L)	Max (µg/L)	Reference Number
As	Southwest Ghana	Permanent large-scale miners	12	14.75	1.62	--	11.41	17.62	[36]
		Casual large-scale miners	12	10.44	1.88	--	9.93	11.59	
		Large-scale miners 0.5 km away from mine	12	8.03	1.75	--	6.49	11.72	
		Large-scale miners 2 km away from mine	12	7.78	1.33	--	5.34	9.77	
		Non-miners 10 m away from mine	12	6.76	1.43	--	5.71	8.91	
As	Tarkwa, Western Region	Tarkwa Mine Workers	15	270.00	160.00	260	34.00	650	[35]
		Tarkwa Non-Mine Workers	17	240.00	160.00	240	43.00	700	
As	Accra, Greater Accra Region	Accra (Control)	4	200.00	69.00	200	120.00	280	[35]
As	Talensi District, Upper East Region	Obuasi, World Bank, & Kejetia,	57	114.52	--	100.21	11.75	354.81	[46]
		Small-scale artisanal miners							
Cd	Tarkwa, Western Region	Tarkwa Mine Workers	15	0.58	0.3	0.62	0.1	1.03	[35]
		Tarkwa Non-Mine Workers	17	0.66	0.42	0.61	0.1	1.88	
Cd	Accra, Greater Accra Region	Accra (Control)	4	0.36	0.06	0.36	0.31	0.42	[35]
Cd	Talensi District, Upper East Region	Obuasi, World Bank, & Kejetia,	57	0.45	--	0.36	0.1	1.32	[46]
		Small-scale artisanal miners							

#### 2.2.4. Certainty Analysis

There is moderate certainty (some scientific publications; plausibility, strength of association, consistency of findings) that ASGM miners and community members across Ghana are exposed to high, and potentially dangerous, levels of toxic metals such as arsenic and cadmium. A number of steps in the mining process (e.g., excavating and crushing ore) facilitate the release of potentially toxic metals into the environment (Figure 2). This has been documented in a number of sites in Ghana and summarized in the Natural Sciences review in this series (e.g., contamination of water and food with relatively high levels of arsenic, cadmium, lead) [8]. Biomarker studies in Ghanaian miners and ASGM community members are beginning to show high exposures. Studies on non-mercury heavy metals in environmental media, biota, and humans tend to be focused in southwest Ghana, leaving a knowledge gap of the status of heavy metals pollution in mining areas in northern Ghana, where officials have mentioned that mining activities are expanding [60].

### 2.3. Occupational Injuries

#### 2.3.1. Causes

Mining is an inherently risky occupation. Hazards are common in large-scale mining operations, which are typically highly organised and well regulated, and are likely to be even more common in ASGM mines, particularly those that are unregistered or illegal. Though, to our knowledge evidence comparing hazards between formal and small-scale operations is not available. Mining involves routine exposures to a wide variety of accident-related, physical hazards, including falls, being struck by objects, extreme temperatures, injuries from power tools and equipment, and lacerations, as well as tunnel collapses resulting from weak ore formations or inadequate trenching and shoring [21]. These hazards result in severe injuries and fatalities, though few data are available to quantify the rates at which injuries and fatalities occur in a typical ASGM facility.

A majority of small-scale miners do not use personal protective equipment (PPE), such as hardhats, safety glasses, gloves and steel toed work boots [21,61]. In a study of 120 miners in Ghana’s Upper East Region, 70% of participants responded that they never use PPE, 5.8% reported “yes” to using protection, while others reported “sometimes” [33]. Similarly, in a study in Cameroon and the Central African Republic, roughly three-quarters of the artisanal miners did not use any PPE, despite working in very remote areas without access to any emergency medical care [62].

Formal safety and health training is often not available to workers and this lack of training and low worker awareness, in addition to financial constraints and inefficient regulatory guidelines and enforcement, are major obstacles to the use of PPE [63]. It is also possible that some mine owners and operators are unwilling to invest in training, PPE or preventive equipment maintenance, viewing worker injuries or even fatalities as an acceptable business expense.

#### 2.3.2. Status and Trends

Very little information is available regarding occupational injuries in ASGM operations. Further, there is no systematic national- or local-scale surveillance of injuries in Ghana or many other countries where ASGM is common. In Geita, a small-scale mining district in Tanzania, mining accidents are responsible for about 11 fatalities annually (denominator unknown), and the main causes of accidents nationwide is thought to be tunnel collapses and underground gases [64]. A survey of 180 artisanal miners in Katanga, Democratic Republic of the Congo, revealed that 72% had experienced at least one accident in the past year (392 total accidents reported), with most being caused by tool handling [65]. Data from the Chief Government Mining Engineer’s office (CGME) in a small-scale mining site in Zimbabwe showed that collapses and machinery/vehicle accidents were each responsible for 25% of the 53 reported mine fatalities in 1997. That study also estimated two unreported deaths per month could occur during illegal mining of closed mines and unsafe alluvial mining conditions [66]. Because the ASGM process is relatively comparable across regions, similar patterns in injuries and accidents can be expected in other areas.

**Figure 6:**
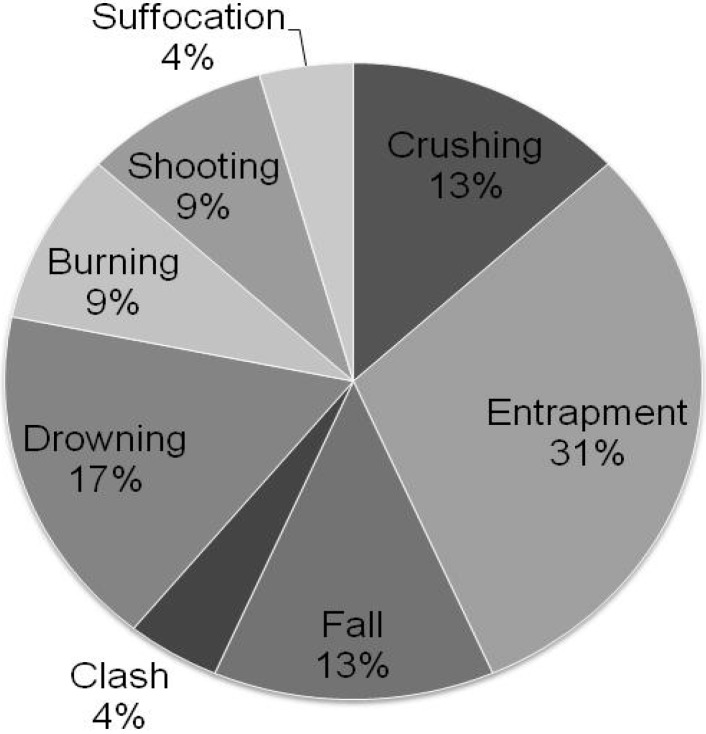
Percentage of injury types recorded in a review of newspaper articles between 9 February 2007 and 11 October 2012 on injuries in small-scale gold mining in Ghana.

To increase understanding of casualties and fatalities among ASGM miners in Ghana, an exploratory research activity utilized media reporting as a source of information [67]. In 2012, we conducted an online search of Ghanaian newspaper articles and found 19 articles reporting 23 separate incidents of accidents and injuries among small-scale gold miners. The first incident found in the search occurred on 9 February 2007, and the last on 11 October 2012. Eight types of incidents were identified from the 23 events (Figure 6, adapted from Brewster, 2013) [67]. Miners trapped in collapsed tunnels was the most common incident type mentioned (7% of 23%, or 30%) followed by drowning (4%, or 17%), and violence (*i.e*., clash and shooting) and falls into mining pits (3%, or 13%, for each of the two types). Collectively, these types of incidents represented nearly 75% of all reported occupational injuries. Other types of events included crushing, burns, firearms injuries, and suffocation. Only three articles mentioned the body part injured, and these included the head, wrist, hand, and upper body. Sixteen of the 23 incidents (70%) were reported from illegal small-scale mines. Twenty of the 23 incidents (87%) resulted in fatalities, with a range of 1 to 18 fatalities per incident, and a total of 76 fatalities, including one Chinese immigrant and one police officer. Note that these media reports cannot be considered representative of national injury and fatality experience among ASGM miners, but these reports nevertheless represent a useful tool for exploring and characterizing injuries and fatalities among miners, and the information may be useful in designing future studies.

With increased growth of ASGM in Ghana, we expect a concomitant increase in the frequency of accidents and injuries. The International Labour Organization [21] estimates that artisanal and small-scale mining (ASM, as opposed to ASGM) operations worldwide experience six to seven times more non-fatal accidents than large-scale operations, with an estimated five to over 20 fatalities annually in Ghana, though these estimates are likely low due to underreporting. Indeed, unsafe working conditions and reports of accidents in Ghanaian ASGM communities have also been documented in the peer-reviewed literature [19,68,69]. For example, a participatory ranking of risks by 46 galamsey miners in the Kumasi Basin revealed life-threatening risks, including tunnel collapses and ground falls near excavation sites. These risks were perceived to be higher than risks posed by mercury use, as accidents were frequent occurrences with adverse outcomes visibly apparent [59].

In the current special issue series, there are two papers which report upon injuries amongst ASGM workers in Ghana. The work by Neitzel *et al.* [70] surveyed injury rates among 72 current ASGM workers in Kejetia, a mining community in the Upper East Region of Ghana (Talensi-Nabdam District) in 2011, and re-surveyed 17 of these workers still engaged in mining activities in 2013 [70]. The 72 workers surveyed in 2011 had worked an average of 6.1–8.1 years in a variety of ASGM activities, including excavating, grinding, sifting, washing, amalgamation, and burning. Injuries were more common among workers with lower levels of education; the fewest injuries were noted among workers who had completed secondary school. Male workers were 12.5 times more likely to be injured than female workers, controlling for education level. Among the 17 workers re-surveyed in 2013, 29% had been injured during mining activities in the past year. Among the seven mining-related injuries that occurred in the past year, the body parts injured included feet, ankles, lower legs, and arms [70]. A second study was conducted by Calys-Tagoe *et al*. [71] in the Tarkwa region of Ghana in which 404 miners were interviewed regarding their injuries over the preceding ten years. The work revealed a total of 121 injury episodes involving 95 miners, with an estimated injury rate calculated to be 5.4 per 100 person years. The most common causes of injuries were falling objects and work-related tools and machinery, and the most affected body parts included the upper and lower limbs and head [71]. Taken together, the above two studies are amongst the first to characterize injuries in ASGM workers, and they both show very high rates of injuries.

#### 2.3.3. Consequences

Though available data are quite sparse, they do suggest that injuries are quite common among ASGM miners. Injuries are known to have a tremendous economic and social impact on the affected workers, as well as on society as a whole [72]. Given the injury rates reported in the few scientific studies and the number of fatalities reported via the media—which undoubtedly misses many, if not most, fatalities that occur in the country—and given that 500,000 to 1 million people are estimated to be employed in ASGM, it is likely that ASGM-related injuries and fatalities represent a substantial health and economic burden [5,73]. Many factors are involved in evaluating the costs of injuries and fatalities, including healthcare costs, lost productivity for the injured worker, lost productivity for the injured worker’s employer, disability costs, and quality of life costs—not to mention the terrible impact on the victim and their families. There are insufficient data available to create even crude estimates of these costs in Ghana.

#### 2.3.4. Certainty Analysis

There is moderate certainty (high plausibility and a number of anecdotes, but scientific peer-review evidence is still limited despite two additional studies performed as part of the current IA) that preventable accidents and injuries occur in ASGM communities in Ghana. Limited scientific studies and a number of newspaper articles have documented accidents and injuries in Ghanaian ASGM communities, with an estimated 25%–50% of people involved in ASGM reporting at least one such occurrence. Plausibility of such events is high given that health and safety issues do not receive adequate support in ASGM mines (e.g., workers are often not provided with appropriate PPE), the working environment is unsafe (e.g., old abandoned concessions, weak geologic formations), and training and technical expertise amongst miners is rudimentary. Plausibility of anecdotal information and media reports is further increased by a large body of evidence indicating high rates of injuries and fatalities among miners in other countries [21,64,65,66,68]. Both active miners and ASGM community members may be affected by this important source of morbidity and mortality. Other sources of data, on occupational injuries and fatalities among Ghanaian ASGM miners could include self-reports collected systematically from miners and summary reports from medical clinics and hospitals in or near mining communities.

### 2.4. Noise and Noise-Related Health Effects

#### 2.4.1. Causes

ASGM involves multiple processes and equipment that can expose miners and nearby communities to noise. In the excavation process, intense noise exposures can occur from use of dynamite, and lower but still potentially important exposures can result from extended use of shovels and picks. Ore processing using generator-powered grinding machines can involve substantial noise exposures, while hand processing using mortar and pestle is likely to produce lower levels. Elevated noise levels are possible in and around ASGM mines throughout the day. The commonly-accepted occupational exposure limit for noise is an eight-hour time-weighted average (TWA) of 85 A-weighted decibels (dBA) [74]. The Ghanaian government has an established regulation limiting noise in industrial settings [75], but no such regulation applies to the mining industry. Even if an applicable regulation were in place, enforcement of existing regulations is often lax in low-income countries [68].

#### 2.4.2. Status and Trends

Virtually no data are available on occupational noise exposures associated with ASGM mining processes. In a study in this special issue, Green et al measured occupational noise exposures among 22 residents of an ASGM mining community (Kejetia, Upper East Region of Ghana) in a 2013 pilot study using personal noise dosimetry and found that, of seven subjects who conducted mining during their monitoring period, all had average noise exposures during their mining activities near or above 85 dBA [76]. The average noise level during mining work was 89.4 dBA, and average noise levels during grinding or crushing operations exceeded 92 dBA. Saunders *et al.* [77] evaluated noise exposures among 59 individuals residing in a Nicaraguan ASGM community. Thirty (51%) subjects were estimated to be exposed to loud noise for ≥40 h per week. A study of a Ghanaian surface gold mine [78] measured sound levels in five areas of the mine, and found that levels in four of these areas exceeded 85 dBA. Another study at a Mexican open-pit copper mine also suggested that high noise exposures were common among miners [79]. There are no published data available on the use of hearing protectors in ASGM mining, though hearing protectors were used <50% of the time they were needed in a South African large-scale gold mine [80]. Our above-mentioned study by Green *et al*. [76] did not observe hearing protector use among any of the participating miners, and no subjects reported use of hearing protection during mining or other noisy activities.

While noise is typically treated as an occupational hazard in the literature, results from our study in Kejetia showed similar noise exposure for miners and non-miners in the community. Of 22 residents surveyed, 50% believed that small-scale mining was the biggest source of noise in their community [76]. Ninety-five percent of the 22 subjects monitored in Kejetia had 24 h noise exposures that exceeded the 70 dBA 24 h limit recommended by the WHO, and differences in 24 h noise exposures were small between miners and non-miners. This suggests that health effects related to noise may not be confined to workers when mining activity is interspersed with residential and commercial areas as exemplified by Rajaee *et al*. [28] in this series.

#### 2.4.3. Consequences

The classically-recognized health effect associated with chronic exposure to noise is noise-induced hearing loss (NIHL) [81]. Loss of hearing has a profound impact on human health and quality of life, and is associated with a wide variety of adverse social, psychological, educational, and occupational outcomes [82]. Worldwide, over 360 million people are estimated to suffer from hearing loss (HL), and HL ranks among the top three most common serious health problems globally [83]. A study of 6428 Ghanaian patients seen for hearing problems at the Komfo Anokye Teaching Hospital in Kumasi found that roughly 90% had hearing loss, and that 8.1% of those cases were NIHL [84]. A study of 252 miners at a large-scale gold mining company in Ghana found that 59 (23%) had NIHL [78]. Among 59 members of an ASGM community in Nicaragua, 21 (35%) had NIHL [77]. Collectively, these results indicate that ASGM miners may be at substantial risk of NIHL.

In addition to NIHL, noise is strongly associated with annoyance, and is increasingly being linked to a host of other health effects, including coronary heart disease, hypertension, sleep disruption, stress, and adverse learning outcomes in children [85,86] due to release of stress-related hormones such as cortisol. No data are available with which to estimate the prevalence of these non-auditory health effects in Ghana, but given the significant impact of these effects, additional research is warranted.

#### 2.4.4. Certainty Analysis

A tremendous body of evidence links occupational noise exposure to hearing loss [81]. There is moderate certainty (high plausibility but few studies) that noise levels in ASGM operations are sufficient to produce NIHL, though the generalisability of the existing measurement data is not well understood. There is also moderate certainty (high plausibility but few studies) that NIHL is common among ASGM miners. Our confidence in these determinations is increased in light of previous analysis of noise exposures and NIHL in large-scale mining operations in high income countries, where high noise exposures and NIHL are quite common [87]. There are insufficient data available to assess the relationship between ASGM noise and non-auditory health effects.

### 2.5. Other Adverse Health Outcomes

#### 2.5.1. Psychosocial Health

As noted in the Social Sciences and Economics review in this series [10], ASGM communities can become thriving economic centres with complementary enterprises springing up [88], but the associated population growth can strain resources and many communities remain impoverished without adequate infrastructure and health services. Poverty and unsanitary living conditions raise concerns for psychosocial stress, inadequate nutrition, infectious disease, and untreated chronic conditions. Tensions and conflict within communities can add to stress while violence brings a risk of injuries and fatalities. The lack of accessible healthcare in many communities compounds these risks. Without financial resources to cope with injuries, disease, and other unexpected issues, poverty increases the risk of “shocks” that decrease well-being temporarily. These shocks can further perpetuate a cycle of poverty [89,90], resulting in inadequate resources to sustain health.

The association between psychosocial stress and adverse health outcomes is well established in the literature. Cardiovascular disease, acute myocardial infarction, inflammation, hypertension, and immune dysregulation, for example, have all been linked with chronic psychosocial stress, which can be related to work, home life, and socioeconomic status [91,92,93,94,95,96]. While evidence for physical health risks of ASGM is increasingly common in the literature [97,98,99], studies on stress in ASGM communities are uncommon. A study by Agyemang [97] found that community members in the Talensi-Nabdam district cited stress as one of many social vulnerabilities they faced. While the study’s aim was to associate environmental degradation with social concerns, it showed that the sources of stressors in the area were actually quite complicated. A pilot study in the same area attempted to quantify stress levels among community members and miners with qualitative and quantitative measures. Twenty-two ASGM miners and community members in Kejetia, a mining community in the Talensi District in the Upper East Region, were asked how often they felt nervous or stressed. Half of the respondents reported feeling nervous or stressed “sometimes”, but women’s responses varied more widely, with 17% answering ‘never’ and 33% answering ‘very often’. Measurements of salivary cortisol, a hormonal biomarker used in stress research, showed only a slight drop in levels from morning to evening, which is a pattern consistent with signs of chronic stress [76].

A case study in the Birim North District cites substance abuse as one of the social problems becoming more of an issue in ASGM communities [19]. Hilson [100] acknowledges that media, government reports, and even scholarly research describes artisanal miners “squandering their monies frivolously on, inter alia, prostitutes, alcohol and narcotics”, yet we are unaware of any data that compare rates of alcohol and drug use in ASGM sites to non-ASGM communities in Ghana.

#### 2.5.2. Nutrition

Sufficient nutrition is critical to child development. Poor maternal nutrition is associated with growth retardation, reduced lung function, impaired cognitive development, and possibly reduced immune function and other adverse health outcomes in offspring [101]. Furthermore, diet diversity has been correlated with educational attainment [102,103,104]. Though nationwide nutrition metrics are improving in most areas, food insecurity and malnutrition occur across Ghana [105,106]; thus, there is reason to believe that these issues are also prevalent in ASGM communities. However, few comparative studies have been conducted between ASGM communities and non-ASGM communities. Migrant households, like many in ASGM communities, may be more likely to be food insecure [107], yet the status of diet and food insecurity has not been well-documented in ASGM settings.

Food security is not always a good indicator of nutrition status, but it can influence physical and psychosocial health outcomes [108,109]. Participants in a study on community concerns in the Talensi-Nabdam district in Ghana's Upper East Region, including local leaders and community members in urban, rural, farming and ASGM communities, identified food insecurity as the top concern related to environmental degradation [97]. In another case study from Kejetia, an ASGM community located in the same region, almost 40% of the 54 household heads surveyed reported being worried about having enough food to feed their family ‘all of the time’ [76]. Dietary data from this community were compared with those from the 2008 Ghana Demographic and Health Survey (DHS), which summarizes data at national and regional levels. Women of child-bearing age surveyed in Kejetia did not appear to be nutritionally deficient in terms of key micronutrients. For example, 100% of women age 15–49 living with a child under three years old consumed foods rich in vitamin A, and 100% consumed foods rich in iron. For both children and women, these numbers are higher in Kejetia than in Ghana as a whole. However, women age 15–49 living with a child under three years old in Kejetia did report lower fruit and vegetable consumption than those nationwide and those in other parts of the Upper East [76].

In addition to concerns about nutritional intake and food insecurity, food safety can be a health concern in ASGM communities. As described extensively in Section 2.1 and Section 2.2, as well as in the Natural Sciences review in this series [8], fish and other edible food items from mining areas have been found to contain mercury and other heavy metals that likely entered the environment because of mining practices. Studies from Ghana and elsewhere have shown high levels of mercury to exist in the hair of miners and community members, suggesting that mercury exposure may be occurring through diet [6]. As outlined in the Natural Sciences review in this series, other food items such as cassava as well as drinking water may be contaminated with a number of toxic metals [8]. Samples of *pito*, a locally-produced millet beer consumed in northern Ghana and other parts of West Africa, had concentrations of aluminium, chromium, manganese, nickel, zinc, arsenic, and lead above WHO drinking water standards. This finding may be due to the *pito* being made with water from mine pits, the excavation of which had likely mobilized these elements [110].

#### 2.5.3. Respiratory and Cardiovascular Health

While biomass cooking smoke is a major concern for respiratory health in rural communities, ASGM miners may also be exposed to respirable crystalline silica in ore, which may exceed 30% crystalline silica in some gold ore dust [111]. Long-term exposure to crystalline silica can cause silicosis, an irreversible pulmonary fibrosis that can exhibit restrictive and obstructive lung disease patterns, which often develop 20 to 45 years after exposure to silica [111]. Miners with silicosis also have accelerated pulmonary function loss [112,113].

In a study of ASG miners in the Upper East Region, elevated abnormal pulmonary function was observed in participants’ forced expiratory volume in the first second (FEV_1_) and the ratio of the FEV_1_ to the forced vital capacity (FVC, or the total volume of air exhaled) [114]. However, pulmonary function measures of FEV_1_, FVC, and the ratio of FEV_1_/FVC were not significantly associated with years involved with mining for participants. Adverse respiratory symptoms of breathlessness and severe breathlessness were more common among ASGM community participants engaging in mining activities, but other symptoms such as chronic bronchitis and shortness of breath were counter-intuitively more common among non-miners. The Healthy Worker Effect may partly explain this phenomenon, as miners with adverse respiratory symptoms may cease mining work or leave mining communities upon developing symptoms. Additionally, a study of ASG miners that are more transient may not show adverse pulmonary function as silicosis and decrements to pulmonary function may not develop for 20 to 45 years after exposures, and may underestimate the impact silica exposure may have on respiratory health (Rajaee, 2015c). The respiratory health of ASG miners has not been studied, but evidence from large-scale miners hints that this may be an area of concern in ASGM communities where there may be concurrent exposures that adversely impact respiratory health.

There is a growing concern for cardiovascular health across Ghana as the prevalence of hypertension increases [115]. In 1973, hypertension was estimated to be prevalent in 4.5% of participants over 16 years from rural Ghanaian villages [116], but in 2010 was estimated to be 20% in rural areas [115]. Exposures and lifestyle factors from ASGM may heighten these risks. Noise, as discussed previously, is associated with coronary heart disease and hypertension [85]. Heavy metals, such as arsenic and mercury have been associated with adverse cardiovascular outcomes. Arsenic is associated with cardiovascular diseases [47]. Mercury has been associated with increases in coronary heart disease, myocardial infarctions, cerebrovascular accidents, cardiac arrhythmias, heart rate variability, atherosclerosis, and renal dysfunction [117,118].

Hair and blood mercury, as proxies for methylmercury exposure, have been associated with increases in systolic and diastolic blood pressure [119,120,121,122]. Urinary mercury, as a proxy for elemental and inorganic mercury exposure, is less understood. Slightly elevated exposures have been associated with decreases in systolic blood pressure [121,123,124], while very high exposures have been associated with increases in systolic and diastolic blood pressure [125]. One study of ASGM community residents from the Talensi District in the Upper East Region found no significant associations between hair and urinary mercury with blood pressure, but observed trends suggestion a non-linear relationship of urinary mercury and blood pressure [126].

#### 2.5.4. HIV/AIDS and Sexual Health

Because of sociocultural and socioeconomic factors described in previous sections and in the Social Sciences and Economics review in this series [10], members of ASGM communities may be especially vulnerable to HIV/AIDS and other sexually transmitted infections (STIs) [127]. Local health authorities in the Birim North District in the Eastern Region reported an increase in teen pregnancy and STIs in the district as galamsey mining activities have proliferated. They attributed the increase to prostitution, substance abuse, influxes of migrants, lack of use of preventive measures, and cultural stigmas surrounding sex [19].

Women may be particularly susceptible given the inadequate access to sex education, healthcare and family planning services in many ASGM communities, and the religious and social stigmas surrounding sex and contraceptive use [19]. Because mining communities are predominantly composed of males with disposable income, economic factors such as underemployment may encourage women to engage in sex work in exchange for money or for jobs at mining sites [19,128,129]. Investigations by UNICEF and Family Health International in Obuasi found that sex work took place in homes, hotels, and brothels [128,129,130], and youth who drop out of school are particularly vulnerable [129]. In a community-wide survey conducted by UNICEF in the same region, 93% of respondents were aware of commercial sex activities in the area, and 67% indicated that these activities were highly visible. As part of the same project, several surveys were carried out among out-of-school youth and commercial sex workers. Among youth out of school but in apprenticeships, 33% had either paid or been paid for sex, compared with 49% of the unemployed youth out of school.

In the baseline survey of commercial sex workers conducted by UNICEF, all respondents knew about HIV/AIDS, could identify at least two modes of transmission, and were aware of condoms. Nearly all (97%) believed that condoms could protect them from getting AIDS, but only 55% of sex workers said that they always succeeded in convincing their clients to use condoms [129]. The survey by National AIDS Control Programme (NACP) found that over 10% of female sex workers in Obuasi had never used a condom and over 48% had experienced STI symptoms in the past 12 months [128]. Aside from STIs, women in ASGM sites face the risk of sexual violence. Women in ASGM sites in Ghana have reported verbal and physical sexual harassment and forced sexual intercourse with men [19].

#### 2.5.5. Water, Sanitation, and Infectious and Vector-Borne Diseases

Lack of sanitation facilities and safe drinking water sources in small-scale gold mining communities may contribute to the spread of infectious diseases. According to one survey in the Upper East Region, 100% of households surveyed in an ASGM community reported the bush or field as their main toilet facility, compared to only 18% of households surveyed throughout Ghana [106,131]. In the same community, 70% of participants used surface water as their main source of drinking water, and 30% used sachet water (*i.e*., water sold in small plastic bags) as their main drinking water source. Other water sources in the community included a tube well/borehole, a dug well, rainwater, and bottled water, but each of these was only ever used by 1.9%, 1.9% 5.6%, and 1.9% of the participants, respectively. Though most survey participants said that mine pit water was never used for drinking, multiple people reported using it for bathing, and researchers witnessed mine pit water being used to brew *pito* [110]. Samples from drinking water sources (including a well at a nearby foreign-operated mine, a stream, mine pits, and *pito*) tested positive for coliform bacteria, indicating possible contamination.

Unreclaimed mine pits can reportedly fill with water to create mosquito breeding grounds [132]. Local health authorities in the Eastern Region of Ghana reported malaria, anaemia, hypertension and diarrhoea as the common diseases that women working in galamsey camps complain of [19]. Though malaria cases were not verified, a 2011 study that asked participants to self-report malaria symptoms and malaria history found 80% of participants from an ASGM community (*n =* 54) reported malaria symptoms and 67% (*n =* 27) from an agricultural community reported malaria symptoms at the time of the survey [133]. No relationship was found between urine mercury levels and self-reported malaria symptoms. Risk factors for malaria were also documented. Bed net usage in the past week, a preventive measure against malaria, was higher in an ASGM community (median: three nights per week) than in a farming community (median: zero nights per week). However, more open water sources suitable for mosquito breeding and more unsealed doors and windows, which allow mosquito entry into the home, were present in the ASGM community: standing water was present within a 25 m radius of the living quarters for 94% of households in the mining community and only 54% of households in the farming community [133]. Crompton, Ventura *et al*., report from a Brazilian ASGM community that although there was no malaria prevalent in the community, those who reported a history of working with mercury were four times more likely to report a malaria infection [134]. A cross-sectional study in 1997 in Para, Brazil did not find a dose-response relationship between mercury exposure and likelihood of prevalent malaria infection, but the authors stated that “there was a possible reduction in acquisition of immunity that may be associated with conditions in gold mining, including mercury exposure” [135]. No data are available on malaria incidence in ASGM sites in Ghana compared to other communities in Ghana. Collecting these data is complicated by the fact that mercury poisoning may be mistakenly treated as malaria [100,136].

In Ghana, the proximity of endemic buruli ulcer communities to mining activity lends itself to speculation on ASGM being a risk factor for this disease. Distance to gold-mining sites has indeed been associated with a higher prevalence of buruli ulcer in the Amansie West District [137]. Increased risk of infection was not associated with direct participation in mining or contact with mine pit water in a case-control study in Ghana [138], but the land use changes that often accompany ASGM, such as streambed disturbance, have been proposed as a mechanism for the spread of buruli ulcer [139].

## 3. Conclusions

A number of health issues have been linked to ASGM in Ghana. Strong evidence suggests elevated mercury exposures in workers and ASGM community members, as well as in nearby biotic and abiotic environmental media. There is also some evidence that ASGM miners and community members are exposed to other heavy metals, such as arsenic and cadmium, mobilized during the mining process. Though data on types, severity and frequency of injuries among ASGM miners in Ghana is limited, studies from other countries, preliminary investigations of injuries in ASGM in Ghana, and reports of unsafe working conditions in Ghana suggest that injuries are common among ASGM miners. Excessive noise exposure, another occupational hazard, has been documented in several ASGM sites in Ghana and may cause hearing loss and other deleterious health effects. There are other health issues of concern for which there are few data but high plausibility, including psychosocial health, cardiovascular, respiratory, and sexual health; nutrition, and water and sanitation.

More in-depth, prospective investigations are needed to further elucidate the nature of the relationships between ASGM and health outcomes in Ghana, and this IA has identified several research areas in need of contributions. However, the existing research on plausible health consequences of ASGM can nevertheless help guide policies and actions to better address the unique challenges ASGM. All of this is particularly warranted and timely given that Ghana recently signed the 2013 UNEP Minamata Convention on Mercury Pollution. This Convention contains specific provisions concerning ASGM (Article 7), and in particular education, outreach and capacity-building initiatives (7.4B), as well as public health aspects (Article 16) of vulnerable groups (women and children; 16.1A) with specific mention of implementation activities (16.1B), health-care services (16.1C), and strengthening of institutional and health professional capacities (16.1D).

In light of this synthesis of extant and emerging data on the health issues associated with ASGM, along with data from the natural sciences [8] and social sciences and economics [10], members of our research team developed response options to address ASGM-related concerns in Ghana using the Delphi method [140]. The data presented here, along with these response options, will provide insight that relevant stakeholders can use to implement enduring solutions to the myriad challenges posed by ASGM in Ghana.

## Appendix

**Table A1:** A summary of mercury concentrations in human urine samples from ASGM miners and non-miners from across Ghana.

Study Location	Population	Sample Size (n)	Mean (µg/L)	SD (µg/L)	Median (µg/L)	Min (µg/L)	Max (µg/L)	Authors
Southwest Ghana	Permanent large-scale miners	12	0.56	0.21	--	0.38	0.76	Abrefah *et al*., 2011 [36]
	Casual large-scale miners	12	0.57	0.14	--	0.4	0.74	
	Large-scale miners 0.5 km away from main site	12	0.51	0.16	--	0.38	0.59	
	Large-scale miners 2 km away from main site	12	0.47	0.12	--	0.36	0.56	
	Non-miners 10 km away from main site	12	0.36	0.11	--	0.29	0.46	
Ankobra & Tano River basins, Western Region	Ankobra basin: Anwiaso (gold mining area)	50	34.2	36	--	1	183	Adimado & Baah 2002 [39]
	Ankobra basin: Sahuma (gold mining area)	50	2.6	1.9	--	0.13	6.96	
	Tano basin: Tanoso (gold mining area)	51	6.4	2.7	--	2	14.3	
	Tano basin: Elubo (gold mining area)	66	7.3	6.9	--	0.02	42.5	
Tarkwa, Western Region	Tarkwa: all	32	4	2.6	3.4	0.5	12	Asante *et al*., 2007 [35]
	Tarkwa: mine workers	15	3.6	2.4	3	0.5	9.4	
	Tarkwa: non-mine workers	17	4.3	2.7	3.6	1.1	12	
Accra, Greater Accra	Accra	4	3.1	1.8	2.8	1.4	5.5	Asante *et al*., 2007 [35]
Dunkwa-on-Offin, Central Region	Small-scale miners: all	40	1.23	0.86	0.86	0.32	3.62	Kwaansa-Ansah *et al*., 2010 [34]
	Small-scale miners: males	30	1.39	0.91	1.23	0.41	3.62	
	Small-scale miners: females	10	0.72	0.43	0.58	0.32	1.84	
	Farmers: all	54	0.69	0.39	0.66	0.075	2.31	
	Farmers: males	41	0.68	0.41	0.61	0.08	2.31	
	Farmers: females	10	0.59	0.31	0.51	0.32	1.22	
Talensi-Nabdam District, Upper East	All participants	120	17	77.3	2.5	0.2	708	Paruchuri *et al*., 2010 [33]
	Males	58	33.1	116.4	4.4	0.2	708	
	Females	62	4.9	8	2	0.3	43.7	
	World Bank	13	1.83	2.3	0.9	0.3	32.9	
	Obuasi	92	15.8	79.7	2.8	0.2	708	
	Kejetia	15	38.9	95.7	3.6	0.5	178.2	

**Table A2:** A summary of mercury concentrations in human hair samples from ASGM miners and non-miners from across Ghana.

Study location	Population	Sample Size (n)	Mean (µg/g)	SD (µg/g)	Median (µg/g)	Min (µg/g)	Max (µg/g)	Authors
Ankobra & Tano River basins, Western Region	Ankobra basin: Anwiaso (gold mining area)	50	1.61	1.33	--	0.15	5.86	Adimado & Baah 2002 [39]
	Ankobra basin: Sahuma (gold mining area)	50	0.62	0.41	--	0.32	2.19	
	Tano basin: Tanoso (gold mining area)	51	4.27	6.26	--	0.06	28.3	
	Tano basin: Elubo (gold mining area)	66	1.21	0.65	--	0.07	3.19	
KNUST, Kumasi (19 students reside in Accra & 18 reside in Kumasi), Ashanti Region, Greater Accra Region	All	37	0.717	0.961	--	0.007	5.535	Anim *et al*., 2011 [43]
	Males	31	0.6	0.545	--	0.007	2.215	
	Females	6	1.417	2.143	--	0.647	5.535	
	Accra students	19	0.551	0.632	--	0.007	2.215	
	Kumasi students	18	0.893	1.213	--	0.009	5.535	
Pra River Basin, Western, Central, and Ashanti Regions	Non-miners	24	1.03	0.77	0.85	0.39	4.13	Donkor *et al*., 2006 [41]
	Small-scale miners	38	1.56	1.22	1.2	0.58	6.5	
Dunkwa-on-Offin, Central Region	Small-scale miners: all	40	2.14	1.53	1.58	0.57	6.07	Kwaansa-Ansah *et al*., 2010 [34]
	Small-scale miners: males	30	2.37	1.59	1.66	0.61	6.07	
	Small-scale miners: females	10	1.72	0.89	1.17	0.57	4.23	
	Farmers: all	54	2.35	1.58	1.67	0.63	7.19	
	Farmers: males	41	2.48	1.66	1.7	0.63	7.19	
	Farmers: females	10	1.43	0.67	1.26	0.71	3.01	
Talensi-Nabdam District, Upper East Region	All participants	100	1.1	3.2	0.4	0	22.9	Paruchuri *et al*., 2010 [33]
	Males	--	1.5	3.5	0.6	0.2	22.9	
	Females	--	0.8	2.9	0.3	0	21.9	
	World Bank	11	0.3	0.2	0.3	0	0.6	
	Obuasi	77	1.1	3.5	0.4	0	22.9	
	Kejetia	12	1.2	1.4	0.6	0.2	4.8	
Not specified	General population	123	0.843	0.557	0.728	0.119	4.14	Voegborlo *et al*., 2010 [42]
